# Pyridyl Methylsulfinyl Benzimidazole Derivatives as Promising Agents against *Giardia lamblia* and *Trichomonas vaginalis*

**DOI:** 10.3390/molecules27248902

**Published:** 2022-12-14

**Authors:** Beatriz Hernández-Ochoa, Víctor Martínez-Rosas, Laura Morales-Luna, Ernesto Calderón-Jaimes, Luz María Rocha-Ramírez, Daniel Ortega-Cuellar, Yadira Rufino-González, Abigail González-Valdez, Roberto Arreguin-Espinosa, Sergio Enríquez-Flores, Rosa Angélica Castillo-Rodríguez, Noemí Cárdenas-Rodríguez, Carlos Wong-Baeza, Isabel Baeza-Ramírez, Saúl Gómez-Manzo

**Affiliations:** 1 Programa de Posgrado en Biomedicina y Biotecnología Molecular, Escuela Nacional de Ciencias Biológicas, Instituto Politécnico Nacional, Mexico City 11340, Mexico; 2 Laboratorio de Inmunoquímica, Hospital Infantil de México Federico Gómez, Secretaría de Salud, Mexico City 06720, Mexico; 3 Laboratorio de Bioquímica Genética, Instituto Nacional de Pediatría, Secretaría de Salud, Mexico City 04530, Mexico; 4 Posgrado en Ciencias Biológicas, Universidad Nacional Autónoma de México, Mexico City 04510, Mexico; 5 Unidad de Investigación en Enfermedades Infecciosas, Hospital Infantil de México Federico Gómez, Dr. Márquez No. 162, Colonia Doctores, Mexico City 06720, Mexico; 6 Laboratorio de Nutrición Experimental, Instituto Nacional de Pediatría, Secretaría de Salud, Mexico City 04530, Mexico; 7 Laboratorio de Parasitología Experimental, Instituto Nacional de Pediatría, Secretaría de Salud, Mexico City 04530, Mexico; 8 Departamento de Biología Molecular y Biotecnología, Instituto de Investigaciones Biomédicas, Universidad Nacional Autónoma de México, Mexico City 04510, Mexico; 9 Departamento de Química de Biomacromoléculas, Instituto de Química, Universidad Nacional Autónoma de México, Mexico City 04510, Mexico; 10 Laboratorio de Biomoléculas y Salud Infantil, Instituto Nacional de Pediatría, Secretaría de Salud, Mexico City 04530, Mexico; 11 CICATA Unidad Morelos, Instituto Politécnico Nacional, Boulevard de la Tecnología, 1036 Z-1, P 2/2, Atlacholoaya, Xochitepec 62790, Mexico; 12 Laboratorio de Neurociencias, Instituto Nacional de Pediatría, Secretaría de Salud, Mexico City 04530, Mexico; 13 Laboratorio de Biomembranas, Departamento de Bioquímica, Escuela Nacional de Ciencias Biológicas, Instituto Politécnico Nacional, Mexico City 11340, Mexico

**Keywords:** antigiardial, antitrichomonal, benzimidazole derivatives, inhibition, enzymes

## Abstract

Protozoan parasites, such as *Giardia lamblia* and *Trichomonas vaginalis*, cause the most prevalent infections in humans in developing countries and provoke significant morbidity and mortality in endemic countries. Despite its side-effects, metronidazole is still the drug of choice as a giardiacidal and trichomonacidal tissue-active agent. However, the emergence of metronidazole resistance and its evolved strategies of parasites to evade innate host defenses have hindered the identification and development of new therapeutic strategies against these parasites. Here, we tested five synthesized benzimidazole derivatives as possible drugs for treating giardiasis and trichomoniasis, probing the bifunctional enzyme glucose 6-phosphate dehydrogenase::6-phosphogluconolactone from *G. lamblia* (GlG6PD::6PGL) and *T. vaginalis* (TvG6PD::6PGL) as a drug target. The investigated benzimidazole derivatives were **H-B2M1**, **H-B2M2**, **H_2_N-BZM6**, **O_2_N-BZM7**, and **O_2_N-BZM9**. The recombinant enzymes were used in inhibition assays, and in silico computational predictions and spectroscopic studies were applied to follow the structural alteration of the enzymes and identify the possible mechanism of inhibition. We identified two potent benzimidazole compounds (**O_2_N-BZM7** and **O_2_N-BZM9**), which are capable of inhibiting both protozoan G6PD::6PGL enzymes and in vitro assays with these parasites, showing that these compounds also affect their viability. These results demonstrate that other therapeutic targets of the compounds are the enzymes GlG6PD::6PGL and TvG6PD::6PGL, which contribute to their antiparasitic effect and their possible use in antigiardial and trichomonacidal therapies.

## 1. Introduction

Infections caused by parasites represent a significant public health crisis in developing countries [1]. In recent years, different strategies have been used in the search for new treatments against protozoan parasites of medical importance, such as *G. lamblia* and *T. vaginalis*. Currently, drug discovery strategies are principally based on identifying essential biomolecules of pharmacological interest which, after inhibiting its activity/function, could be lethal to the parasite. Parasite metabolic pathways provide an attractive target for drug development and are, therefore, of particular interest [2]. For example, it has been shown that the disruption of the glycolysis via the inhibition of triosephosphate isomerase enzyme (TIM) affects the viability of *G. lamblia*, suggesting that this enzyme is identified as a pharmacological target [3,4]. A similar effect was observed by several compounds that selectively inhibit the TIM of *T. vaginalis* [5,6]. Additionally, the repurposing of drugs is an attractive pharmacological strategy for new therapeutics [7].

Over the years, benzimidazole compounds have received considerable attention in chemistry medicinal owing to their wide biological activities and diverse therapeutic applications. Thus, benzimidazole derivatives are crucial structural scaffolds found in diverse libraries of biologically active compounds which are therapeutically useful agents in drug discovery and medicinal research. Numerous compounds containing benzimidazole moieties have been reported to exhibit diverse biological and pharmacological properties, including analgesic, antibacterial, anticancer, and antiparasitic activities.

**Figure 1:**
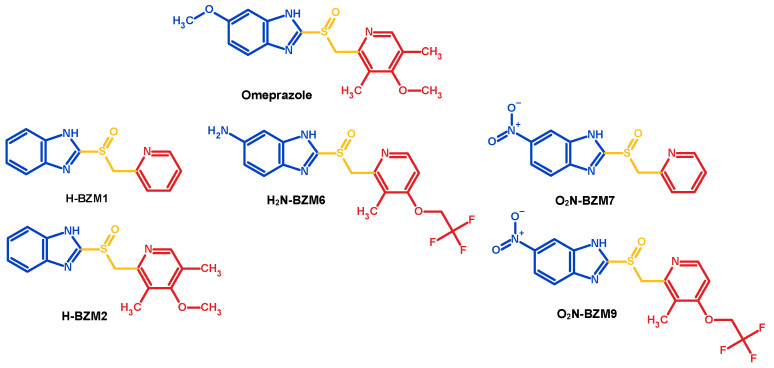
Chemical structure of pyridyl methylsulfinyl benzimidazole compounds analogous to omeprazole; the benzimidazole and pyridine core are shown in blue and red color.

In this scenario, the commercial drug omeprazole (derived from pyridyl methylsulfinyl benzimidazole) has been suggested as an antigiardial compound, since it selectively inactivates the triosephosphate isomerase of *G. lamblia* (GlTIM) by chemical modification of cysteine residues, which leads to the parasite’s death [8]. In addition, some other proton pump inhibitors (PPIs) are reported to be active against *Giardia* trophozoites in vitro in the range of effectivity for albendazole [4,9,10]. On the basis of the above, we previously designed and synthesized five compounds analogous to omeprazole (Figure 1), 2-[(pyridin-2-yl)methanesulfinyl]-1*H*-benzimidazole (**H-BZM1**), 2-[(4-methoxy-3,5-dimethylpyridin-2-yl)methanesulfinyl]-1*H*-benzimidazole (**H-BZM2**), 2-{[3-methyl-4-(2,2,2-trifluoroethoxy)pyridin-2-yl]methanesulfinyl}-1*H*-benzimidazol-6-amine (**H_2_N-BZM6**), 6-nitro-2-[(pyridin-2-yl)methanesulfinyl]-1*H*-benzimidazole (**O_2_N-BZM7**), and 2-{[3-methyl-4-(2,2,2-trifluoroethoxy)pyridin-2-yl]methanesulfinyl}-6-nitro-1*H*-benzimidazole (**O_2_N-BZM9**) [11], whose difference lies in different substituent groups linked to the benzimidazole and pyridine ring and enhances the antigiardial activity of omeprazole through the inhibition of the GlTIM enzyme. Notably, the previously analyzed compounds with the most antigiardial activity were **O_2_N-BZM7** and **O_2_N-BZM9**. These compounds also showed a low cytotoxicity in Caco-2 and HT29 cell lines; however, whether these compounds have another protozoan or protein target needs to be evaluated. 

The aforementioned results allowed the optimization of the antigiardial activity of omeprazole-like compounds through chemical modifications and, thus, identification of two compounds with greater antigiardial activity and with the potential to inhibit GlTPI. However, there is increasing evidence that a drug interacts with various molecular targets. For example, it has been reported that omeprazole binds to multiple proteins and can form highly stable complexes that are not dependent on disulfide linkages between the drug and protein targets [12]. Therefore, it was of our interest to study the bifunctional enzyme glucose 6-phosphate dehydrogenase::6-phosphogluconolactone (G6PD::6PGL) as a possible drug target. This enzyme is involved in the pentose phosphate pathway and provides the parasite with NADPH and ribose molecules, which are essential for the survival of most protozoans. They are also a possible biomolecule target for benzimidazole compounds, which could contribute to giardiacidal and trichomonicidal activities through the inhibition of G6PD::6PGL.

On the basis of the above, in this study, we analyzed the effect of **H-BZM1**, **H-BZM2**, **H_2_N-BZM6**, **O_2_N-BZM7**, and **O_2_N-BZM9** compounds on the enzyme G6PD::6PGL from *G. lamblia* (GlG6PD::6PGL) and *T. vaginalis* (TvG6PD::6PGL) to demonstrate the ability of the compounds to inactivate these enzymes. We found that compounds **O_2_N-BZM7** and **O_2_N-BZM9** have the most promising effects against giardiasis and trichomoniasis since they inactivate the G6PD::6PGL enzymes of both parasites, alter their structures, and efficiently cause the death of *G. lamblia* and *T. vaginalis* trophozoites.

## 2. Results and Discussion

### 2.1. In Vitro Screening of GlG6PD:6PGL and TvG6PD::6PGL Inactivation with Benzimidazole Compounds

Previously, it was reported that omeprazole and PPI analogs exhibit antigiardial activity because these compounds act as inhibitors of the glycolytic enzyme GlTIM by covalently binding to cysteine residues [4,10]. In this sense, Hernandez-Ochoa et al. [11], with the aim of enhancing the antigiardiasis activity of omeprazole, synthesized and probed the antigiardial effect of five new pyridyl methylsulfinyl benzimidazole compound omeprazole analogs, named **H-BZM1**, **H-BZM2**, **H_2_N-BZM6**, **O_2_N-BZM7**, and **O_2_N-BZM9**. Thus, it was determined that one of the pharmacological targets of these compounds is the glycolytic enzyme GlTIM, although it cannot be the only target. Therefore, it is in our interest to know if these compounds with inhibitory capacities in GlTIM also have the capability to inhibit the fused recombinant G6PD::6PGL enzymes from *G. lamblia* and *T. vaginalis*. 

It is important to mention that these compounds are structural analogs to proton pump inhibitors; they maintain the core of a substituted benzimidazole ring joined to a substituted pyridine linked by a methylsulfinyl chain and, thus, they are chiral compounds. In this work, all the assays were carried out with the racemic mixture of each one of the compounds (R and S enantiomers). To determine the inhibitory effect of benzimidazole compounds on the activity of the fused enzymes, a general assay was performed using 400 μM of each compound. Table 1 shows the inactivation results for both enzymes; we observed that these compounds showed an inhibitory activity in the enzymes to different degrees of inactivation. The complete activity of the GlG6PD::6PGL enzyme was eradicated by four compounds (**H-BZM2**, **H_2_N-BZM6**, **O_2_N-BZM7**, and **O_2_N-BZM9**), while the **H-BZM1** compound inhibited only a 62% activity. This demonstrates that the fused enzyme GlG6PD::6PGL is totally inactivated with benzimidazole compounds. On the other hand, the enzyme TvG6PD::6PGL was only inhibited with **O_2_N-BZM7** and **O_2_N-BZM9** compounds by 95% and 72%, respectively, while the other three compounds inhibited the enzyme activity by less than 50%.

Our results support previous research on GlTIM inhibition, where the best compounds with antigiardial activity were **H-BZM2**, **O_2_N-BZM7**, and **O_2_N-BZM9** [11]. Therefore, we decided to use **H-BZM2**, **O_2_N-BZM7**, and **O_2_N-BZM9** compounds for further assays on the fused enzyme GlG6PD::6PGL. On the other hand, **O_2_N-BZM7** and **O_2_N-BZM9** compounds were used with the TvG6PD::6PGL enzyme for presenting and inhibition percentage superior to 70%.

**Figure 2:**
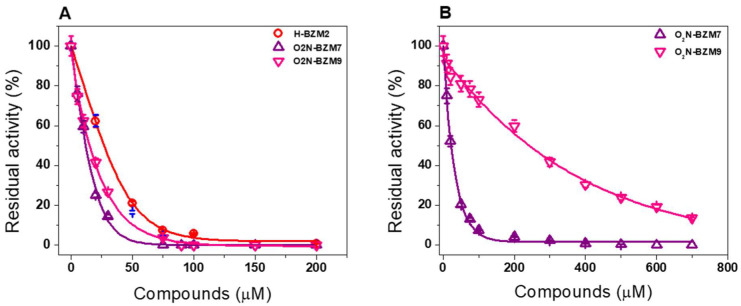
G6PD::6PGL enzyme inactivation assays with compounds. Effect of compounds **H-BZM2**, **O_2_N-BZM7**, and **O_2_N-BZM9** on (**A**) GlG6PD::6PGL and (**B**) TvG6PD::6PGL. The protein was adjusted to a concentration of 0.2 mg/mL and incubated with increasing concentrations of each compound for 2 h at 37 °C. At the end of the incubation time, residual activity was measured. Results are shown as the mean values of three independent tests.

To calculate the concentration of compound needed to inactivate 50% of the activity of the GlG6PD::6PGL and TvG6PD::6PGL enzymes, inactivation assays were performed using appropriate concentrations of the compounds. Figure 2A shows the residual activity of the GlG6PD::6PGL enzyme after incubation with the benzimidazole compounds. It was observed that, as the concentration of each compound increases, the residual activity of the enzyme decreases. In fact, the exposure of the GlG6PD::6PGL enzyme to **H-BZM2**, **O_2_N-BZM7**, and **O_2_N-BZM9** induced the abolition of enzyme activity in a similar way. In this regard, the enzyme lost 100% of its activity when incubated with 100 µM of **H-BZM2** and **O_2_N-BZM9**, whereas **O_2_N-BZM7** seems to be more effective and requires a lower concentration (60 μM) to eradicate enzyme activity. We also evaluated the IC_50_ and found that **O_2_N-BZM7** was more effective as the concentration required to reduce GlG6PD::6PGL enzyme activity by 50% was 11 µM, whereas, for **H-BZM2** and **O_2_N-BZM9**, the IC_50_ values were 24 μM and 15 μM, respectively (Figure 2A).

With respect to TvG6PD::6PGL enzyme, **O_2_N-BZM7** showed an IC_50_ value of 22 μM, while the compound of **O_2_N-BZM9** had an 11-fold higher IC_50_ value (240 μM) (Figure 2B). It is interesting to note that these two compounds have a nitro group (NO_2_) in position 5 of the benzimidazole ring, suggesting that the NO_2_ is an essential cause of the potent inhibitory effect on enzymes. On the other hand, compound **O_2_N-BZM7** has only hydrogen atoms in the pyridine ring, whereas **O_2_N-BZM9** has a methyl group and a trifluoroethoxy group, suggesting that these large groups probably affect the interaction of this compound with the enzyme. A similar effect was also observed for the enzyme GlG6PD::6PGL, where compound **O_2_N-BZM7** showed a lower IC_50_ value than compound **O_2_N-BZM9**.

Lastly, it is interesting to mention that **H-BZM2**, **O_2_N-BZM7**, and **O_2_N-BZM9** compounds presented a better inactivation in the G6PD::6PGL-fused enzyme from *G. lamblia* regarding the TIM enzyme from the same organism, where IC_50_ values of 37 µM, 12 µM, and 20 µM were reported, respectively [11]. Moreover, **O_2_N-BZM7** and **O_2_N-BZM9** compounds showed a better inactivation profile in the fused enzyme from *G. lamblia* regarding TvG6PD::6PGL, suggesting a probable selectivity inhibition of these compounds in the fused enzyme of *G. lamblia* with respect to the enzyme of *T. vaginalis*.

### 2.2. Second-Order Rate Constant (k_2_) of Selected Compounds

**Figure 3:**
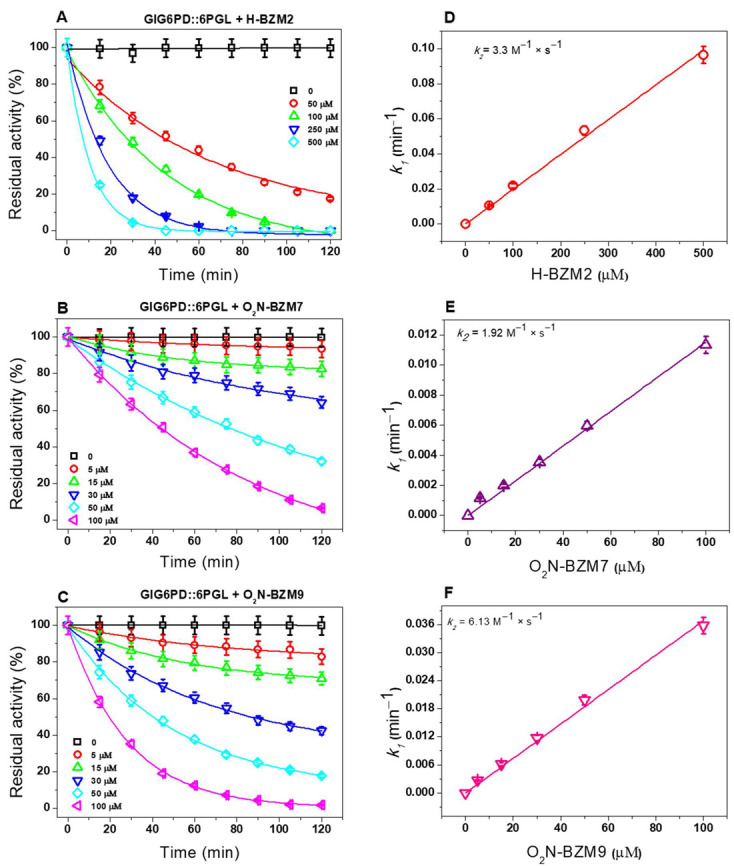
Inactivation assays of G6PD::6PGL enzyme form *G. lamblia* with **H-BZM2**, **O_2_N-BZM7**, and **O_2_N-BZM9**. (**A**–**C**) Pseudo-first-order rate constants (*k_1_*) for each compound were calculated by fitting the data to the exponential decay equation *A_R_ = A_0_ e^−kt^*, where *A_0_* is the initial activity value. All the experiments were performed in triplicate; the bars represent the standard error. (**D**–**F**) Second-order inactivation rate constants (*k_2_*) were obtained by plotting *k_1_* values against compound concentrations and fitted to a linear regression model. All experiments were performed in triplicate; standard errors were less than 5%.

To determine the reactivity of chemical compounds on the GlG6PD::6PGL and TvG6PD::6PGL enzymes, second-order inactivation rate constants (*k_2_*) were calculated. The *k_2_* constant represents the rate of formation of the enzyme–inhibitor complex. In Figure 3A–C, we can see that the inactivation of GlG6PD::6PGL enzyme by the chemical compounds exhibits the behavior of a pseudo-first-order inactivation. Thereafter, the calculated *k_1_* values were plotted versus the chemical compound concentrations, and the second-order inactivation rate constant (*k_2_*) was calculated. The calculated *k_2_* values for **H-BZM2**, **O_2_N-BZM7**, and **O_2_N-BZM9** were 3.3, 1.9, and 6.1 M^−1^·s^−1^, respectively (Figure 3D,E,F). These results suggest that the **O_2_N-BZM9** compound rapidly forms an inhibitor–enzyme complex, followed by **O_2_N-BZM7** and **H-BZM2**. Moreover, these results show that the enzyme G6PD::6PGL of *G. lamblia* is also an efficient target of these compounds, which potentially contributes to their antigiardial activity since they effectively form an inhibitor–enzyme complex with the GlG6PD::6PGL enzyme.

Previously, it was reported that **O_2_N-BZM7** and **O_2_N-BZM9** compounds presented *k_2_* values of 3.2 M^−1^·s^−1^ and 2.8 M^−1^·s^−1^ over the inactivation of GlTIM enzyme [11]. Therefore, these results demonstrate the great ability of the **O_2_N-BZM7** and **O_2_N-BZM9** compounds to form an enzyme–inhibitor complex with the GlG6PD::6PGL and TIM enzymes from *G. lamblia*, which contributes to the antigiardial effect of the compounds. 

**Figure 4:**
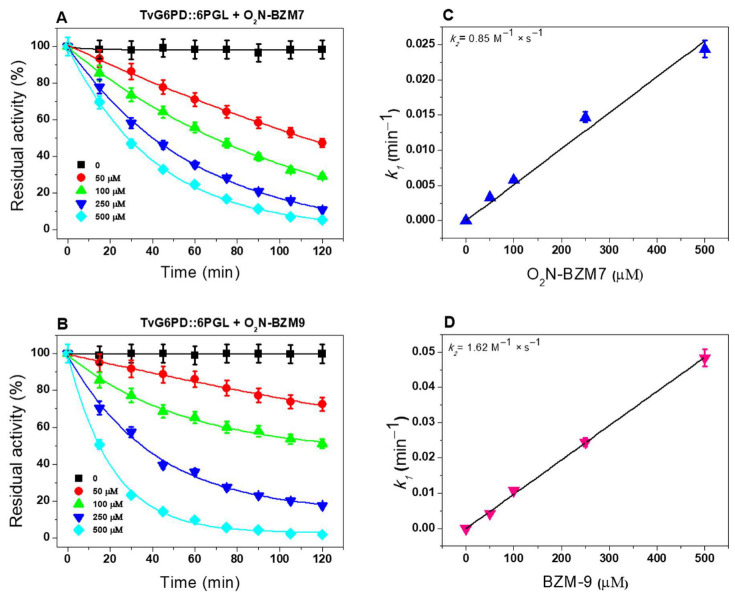
Inactivation assays of the enzyme G6PD::6PGL of *T. vaginalis* with **O_2_N-BZM7** and **O_2_N-BZM9**. (**A**,**B**) Pseudo-first-order rate constants (*k_1_*) for each compound were calculated by fitting the data to the exponential decay equation A_R_ = A_0_ *e^−kt^*, where A_0_ is the initial activity value. (**C**,**D**) Second-order inactivation rate constants (*k_2_*) were obtained by plotting *k_1_* values against compound concentrations and fitted to a linear regression model. All experiments were performed in triplicate; standard errors were less than 5%.

Regarding the TvG6PD::6PGL enzyme, the highest reactivity was shown by the compound **O_2_N-BZM9** with a *k_2_* value of 1.62 M^−1^·s^−1^ (Figure 4B,D), which is twice the value determined for **O_2_N-BZM7** (0.85 M^−1^·s^−1^) (Figure 4A,C). It is interesting to note that, when we compared the *k_2_* values for TvG6PD::6PGL with the *k_2_* value for the enzyme GlG6PD::6PGL (Table 2), **O_2_N-BZM7** and **O_2_N-BZM9** compounds formed an enzyme–inhibitor complex faster with the fused enzyme of *G. lamblia* compared to the enzyme of *T. vaginalis*, which suggests a species-specific inhibition of the compounds toward the enzyme GlG6PD::6PGL.

Lastly, it is important to mention that the evaluated **O_2_N-BZM7** and **O_2_N-BZM9** compounds show a high inhibitory potential in the GlG6PD::G6PD enzyme compared to the results previously reported by Martínez-Rosas et al. [13]. In that report, the authors investigated the impact of an in-house chemical library of 55 compounds on the activity of the fused TvG6PD::6PGL protein and found four compounds—JMM-3, CNZ-3, CNZ-17, and MCC-7—that inhibited the TvG6PD::6PGL protein; however, the calculated *k_2_* values were 0.33, 0.66, 0.38, and 0.26 M^−1^·s^−1^, respectively.

### 2.3. Spectroscopic and Chromatographic Characterization

To determine the mechanism probability of inactivation by the chemical compounds on the fused G6PD:.6PGL from *G. lamblia* and *T. vaginalis*, alterations in secondary, tertiary structure, and quaternary structure were determined in the presence of **O_2_N-BZM7** and **O_2_N-BZM9** chemical compounds since they presented a better inactivation profile in both proteins, with lower IC_50_ values, and rapidly formed the inhibitor-enzyme complex.

#### 2.3.1. Circular Dichroism Experiments

**Figure 5:**
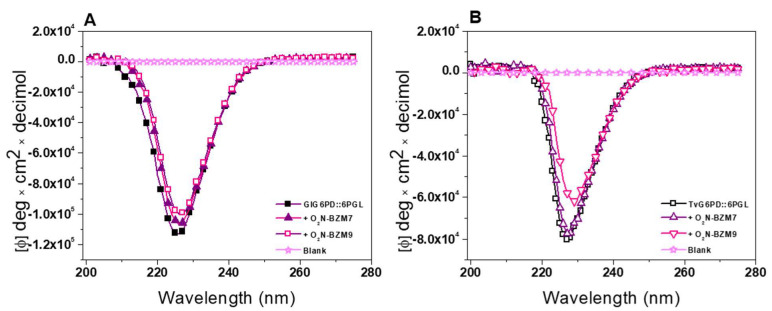
Spectroscopic characterization of enzymes. (**A**) Far-ultraviolet circular dichroism (CD) spectra of the enzyme GlG6PD::6PGL and (**B**) TvG6PD::6PGL. Changes in molar ellipticity were monitored by circular dichroism. The protein was adjusted to 0.5 mg/mL in 50 mM phosphate buffer and incubated with the benzimidazole compounds at the IC_50_ concentration of each inhibitor for 2 h at 37 °C before measuring. The results shown are representative of triplicate experiments.

Firstly, we examined changes in the secondary structure by the circular dichroism (CD) signal of GlG6PD::6PGL and TvG6PD::6PGL proteins in the absence of the benzimidazole compound, which was used as a control. Then, CD spectra of the enzymes were determined after the incubation with the compounds at the IC_50_ concentration. As seen in Figure 5, no significant changes were observed in the presence of the compounds in the fused G6PD::6PGL proteins from *G. lamblia* and *T. vaginalis*. The **O_2_N-BZM7** and **O_2_N-BZM9** chemical compounds did not induce alterations of the secondary structure, as indicated by the similarity of the CD spectra of the GlG6PD::6PGL enzyme without compounds. On the basis of these results, we suggest that the loss of activity can be given at the level of tertiary and/or quaternary structure. Lastly, it is interesting to mention that, in the study carried out by Hernández-Ochoa et al. (2020) [11] with the same compounds in the enzyme TIM from *G. lamblia*, it was found that these same compounds showed a negative effect on the secondary structure of GlTIM. This difference in the alterations in the secondary structure could indicate that the inactivation mechanism is different from that previously reported in the GlTIM, where the sulfur atom of the benzimidazole ring forms a covalent bond with the cysteine residues present in the TIM protein from *G. lamblia* [11].

#### 2.3.2. Intrinsic Fluorescence Assays

**Figure 6:**
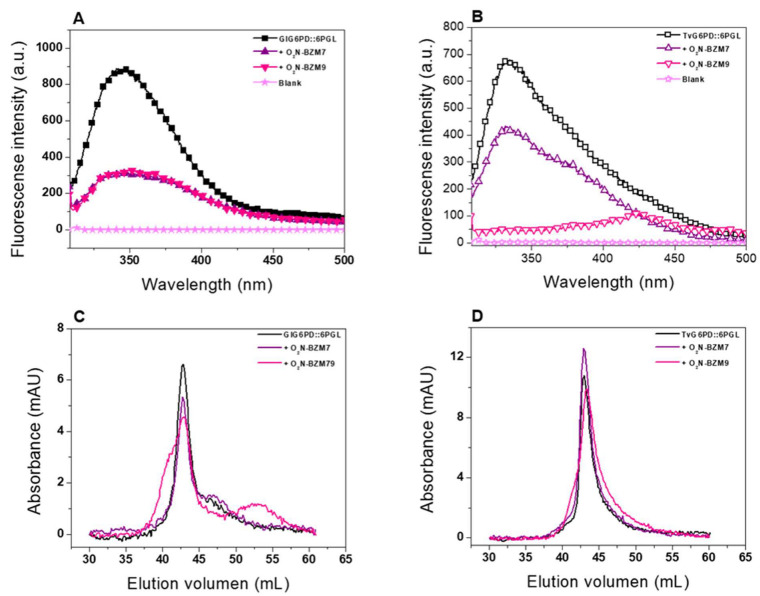
Spectroscopic and chromatographic characterization. (**A**) Intrinsic fluorescence emission spectra of GlG6PD::6PGL enzyme and (**B**) TvG6PD::6PGL in the absence and presence of the benzimidazole compounds. The values obtained from the buffer without protein corresponded to the blank and were subtracted from the spectra obtained with protein. Data are presented as the mean of three experiments. (**C**) Gel filtration chromatography of GlG6PD::6PGL and (**D**) TvG6PD::6PGL in the absence or presence of the compounds. The enzymes (0.5 mg/mL) were incubated at 37 °C for 2 h with each of the benzimidazole compounds and then loaded onto a Sephacryl TMS-200 HR HiPrepTM 16/60 column.

Changes in the tertiary structure and global stability of the fused G6PD::6PGL protein from *G. lamblia* and *T. vaginalis* were evaluated by monitoring the intrinsic fluorescence properties of the eight tryptophan residues contained in the GlG6PD::6PGL/monomer and the nine tryptophan residues of the TvG6PD::6PGL/monomer. Regarding fused G6PD::6PGL from *G. lamblia*, we observed that the two compounds presented a lower intrinsic fluorescence intensity of G6PD::6PGL protein with respect to the control (Figure 6A). The intrinsic fluorescence of the native GlG6PD::6PGL protein without inhibitors showed a peak at 344 nm with a maximum intensity of 874 arbitrary units (a.u.), while both **O_2_N-BZM7** and **O_2_N-BZM9** chemical compounds showed the highest negative effects with a maximum fluorescence intensity of 329 a.u., indicating a reduction of 62% compared to the control. These results are in agreement with those previously observed for the TIM enzyme from *G. lamblia*, where a decrease in fluorescence intensity with these two compounds was also observed [11]. 

Regarding the intrinsic fluorescence intensity of fused TvG6PD::6PGL protein from *T. vaginalis*, we observed that **O_2_N-BZM9** decreases 84% of intrinsic protein fluorescence (102 a.u.) with respect to the native protein (673 a.u.), while **O_2_N-BZM7** showed a maximal fluorescence intensity of 415 a.u, indicating a reduction of 30% compared to the TvG6PD::6PGL enzyme without compounds (Figure 6B). Interestingly, a redshift was observed in the presence of **O_2_N-BZM9**, suggesting the exposure of the solvent to previously buried hydrophobic regions. Moreover, the two benzimidazole compounds affect the fused G6PD::6PGL proteins of the native parasite, indicating that the compounds caused a rearrangement in the microenvironment of the tryptophan residues after the incubation with the compounds.

Lastly, we evaluated the changes in the quaternary structure using size exclusion chromatography. As seen in Figure 6C,D, no significant changes were observed in the retention time in the fused G6PD::6PGL protein from *G. lamblia* and *T. vaginalis* in the absence and presence of compounds, indicating that both enzymes were eluted as single peaks with retention volumes corresponding to native tetramers. These results suggest that the inactivation of fused proteins by benzimidazole compounds does not alter the dimeric structure of the enzyme and induces local modifications of the tridimensional structure instead of global alterations.

The spectroscopic and chromatographic results indicate that benzimidazole compounds alter the tridimensional structure of the proteins, resulting in a loss of catalytic activity in both fused enzymes. Furthermore, these results are correlated with the loss of catalytic activity and are in accordance with biochemical assays, in which the benzimidazole **O_2_N-BZM9** compound formed the enzyme–inhibitor complex faster than **O_2_N-BZM7**.

### 2.4. Molecular Docking Study

**Figure 7:**
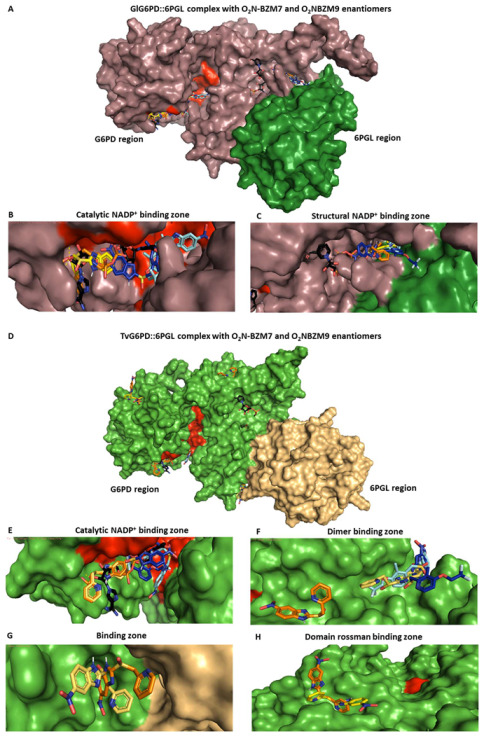
Prediction of the interaction zones between the **O_2_N-BZM7** and **O_2_N-BZM9** enantiomers and the GlG6PD::6PGL and TvG6Pd::6PGL proteins. (**A**) Closer view of the binding sites of **O_2_N-BZM7** and **O_2_N-BZM9** with GlG6PD::6PGL, (**B**) catalytic NADP^+^ binding, and (**C**) structural NADP^+^ binding site zones. (**D**) Closer view of the binding sites of **O_2_N-BZM7** and **O_2_N-BZM9** with TvG6PD::6PGL, (**E**) catalytic NADP^+^ binding, (**F**) dimer binding zone, (**G**) binding zone, and (**H**) domain Rossman binding zone. The NADP^+^ molecule is shown in black, and (**R**)-**O_2_N-BZM7,** (**S**)-**O_2_N-BZM7,** (**R**)-**O_2_N-BZM9,** and (**S**)-**O_2_N-BZM9** are shown in orange, yellow, light blue, and dark blue, respectively.

Since the complete 3D structure of G6PD::6PGL of *G. lamblia* and *T. vaginalis* is still unknown, the models generated by Morales-Luna et al. 2018 [14] and Martinez-Rosas et al. [13] were used to identify probable interaction zones for compounds, and the (R) and (S) enantiomers of the **O_2_N-BZM7** and **O_2_N-BZM9** compounds were used for the blind docking analysis. The docking performed on the entire surface of the homodimer indicated that both (R) and (S) enantiomers of the compounds specifically bind to the G6PD domain of the fused enzyme. They do not bind near any cysteine residue present in this domain, and both (R) and (S) enantiomers of each compound have almost the same binding affinity. Representative binding sites for each compound are shown in Figure 7A–H. This analysis allowed us to know the probable binding zones of the compounds; however, as has been reported, the molecular coupling method is not reliable to accurately predict the preference of the targets for the R or S enantiomers [15].

It is interesting to note that the compounds do not bind near any cysteine residue of the G6PD domain; these results are in concordance with the previously reported by Cartee and Wang [12], where they identified the binding of omeprazole to protein targets by monoclonal antibodies and concluded that omeprazole binds to multiple proteins and is capable of forming highly stable complexes that are not dependent on disulfide linkages between the drug and protein targets. 

**Figure 8:**
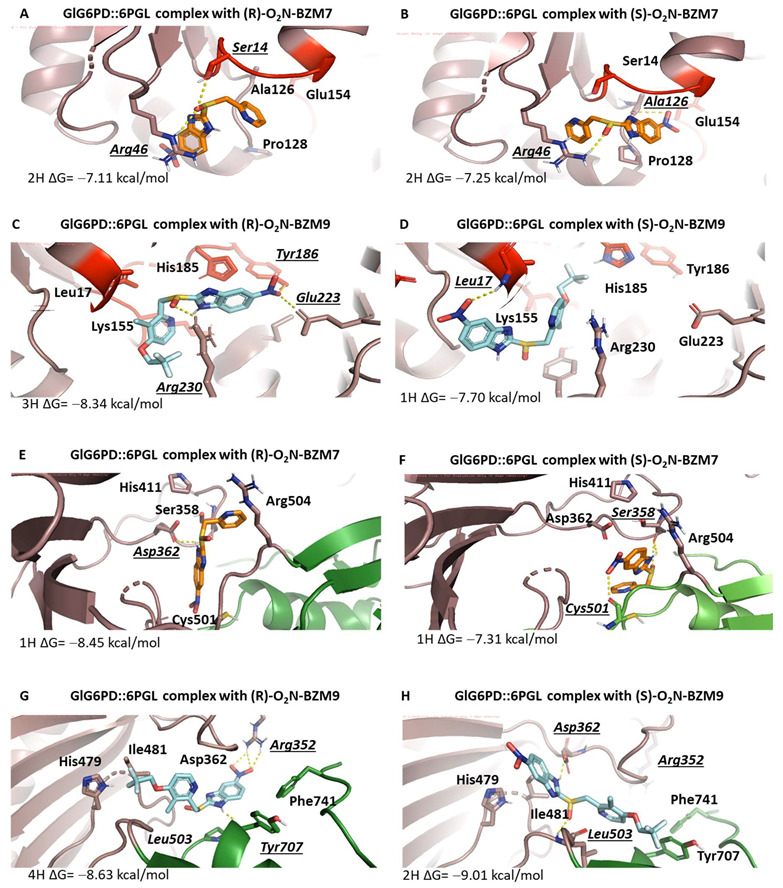
Interaction zones of the chemical compounds **O_2_N-BZM7** and **O_2_N-BZM9** on the GlG6PD::6PGL protein. (**A**) Interaction between the residues of the NADP^+^ catalytic site of GlG6PD::6PGL in the presence of (**R**)-**O_2_N-BZM7**, (**B**) (**S**)-**O_2_N-BZM7,** (**C**) (**R**)-**O_2_N-BZM9,** and (**D**) (**S**)-**O_2_N-BZM9**. (**E**) Interaction between the residues of the NADP^+^ structural site of GlG6PD::6PGL in the presence of (**R**)-**O_2_N-BZM7**, (**F**) (**S**)-**O_2_N-BZM7**, (**G**) (**R**)-**O_2_N-BZM9**, and (**H**) (**S**)-**O_2_N-BZM9**. The **O_2_N-BZM7** and **O_2_N-BZM9** compounds are shown in orange and light blue, respectively. H-bonds are represented in italic and underlined.

The docking analysis revealed a pocket of interaction in both enzymes for the two compounds, whose zone is near the catalytic site. In this pocket, interactions between the compounds and amino acids that participate in the correct positioning of catalytic NADP^+^ were identified. In the case of the compound (**R**)-**O_2_N-BZM7** and the enzyme GlG6PD::6PGL, it was identified that two H-bonds are formed between the sulfoxide group of the compound and the amino acids Arg46 and Ser14; the amino acid Ser14 is part of the hexapeptide conserved (GxxGGDLA) in the G6PD’s enzymes, whose function is the correct positioning of the catalytic NADP^+^ [16] (Figure 8A), and the most stable conformer showed a ΔG of −7.11 kcal/mol. Regarding (**R**)-**O_2_N-BZM9**, it showed a binding energy of −8.34 kcal/mol and formed one H-bond between the sulfoxide group of the compound and Arg230, as well as two H-bonds between the nitro group, and Glu223 and Tyr186 (Figure 8C). This last amino acid is found in the conserved sequence 182-RIDHYLGKE-190 (the amino acid number corresponding to GlG6PD::6PGL sequence) [17]. Additionally, Glu233 is important for G6P binding and defining the shape of the binding site [18]. Another zone of interaction in GlG6PD::6PGL was near the structural NADP^+^ binding site, where (**R**)-**O_2_N-BZM7** showed a ΔG of −8.45 kcal/mol and formed one H-bond with the sulfoxide group and Asp362 (Figure 8E), while (**R**)-**O_2_N-BZM9** formed three H-bonds with the nitro group and Arg352 and one H-bond with benzimidazole ring and Tyr707, with an energy binding of −8.63 kcal/mol (Figure 8G). Similar results were found with the (S) enantiomer of **O_2_N-BZM7** and **O_2_N-BZM9** compounds (Figure 8B,D,F,H). These results show that **O_2_N-BZM9** has a more favorable binding energy than **O_2_N-BZM7** in both zones, and, according to the determined inhibition constant *k_2_*, **O_2_N-BZM9** (6.13 M^−1^·s^−1^) is able to inhibit the enzyme faster than **O_2_N-BZM7** (*k_2_* of 1.92 M^−1^·s^−1^). In addition, the most stable conformer in the catalytic pocket is positioned very close to the nicotinamide ring of NADP^+^, which is the receptor for electrons and protons in the catalytic reaction, in such a way that the presence of **O_2_N-BZM9** more efficiently decreases the activity of the enzyme than **O_2_N-BZM7**.

**Figure 9:**
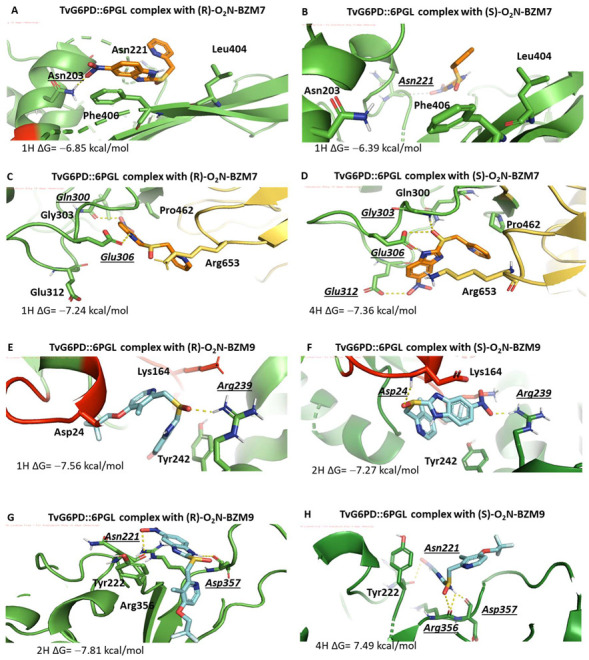
Interaction zones of the chemical compounds **O_2_N-BZM7** and **O_2_N-BZM9** in the TvG6PD::6PGL protein. (**A**) Interaction zone near the structural NADP^+^ site (**R**)-**O_2_N-BZM7**, (**B**) (**S**)-**O_2_N-BZM7,** and (**C**) the 6PGL domain of TvG6PD::6PGL with the compound (**R**)-**O_2_N-BZM7** and (**D**) (**S**)-**O_2_N-BZM7**. (**E**) Close view in the NADP^+^ catalytic site of the (**R**)-**O_2_N-BZM9** and (**F**) (**S**)-**O_2_N-BZM9**. (**G**) Interaction of the (**R**)-**O_2_N-BZM9**, and (**H**) (**S**)-**O_2_N-BZM9** compound with residues close to the structural NADP^+^ site. The **O_2_N-BZM7** and **O_2_N-BZM9** compounds are shown in orange and light blue, respectively. H-bonds are represented in italic and underlined.

The docking of TvG6PD::6PGL and (**R**), (**S**)-**O_2_N-BZM7** revealed four probable zones of interaction (Figure 7D), whose the most stable zone corresponds to the region where the dimer is formed with a binding energy of −6.58 kcal/mol, where 44% of the conformers were found and the formation of a hydrogen bond between the nitro group and Asn203 occurred (Figure 9A). The second most probable zone is found in the 453-LKDKYPEI-460 loop; this loop is close to the 6PGL domain, with a binding energy of −7.24 kcal/mol, and 22% of the conformers were found in this zone (Figure 9C). The third zone corresponds to a zone close to the Rossman-type domain of the G6PD monomer with 19% of the population of conformers (a closer view is seen Figure 7H), and the zone where 16% of the conformers were bound corresponds to the catalytic NADP^+^ binding site (an overview is shown in Figure 7E). On the other hand, docking with (**R**)-**O_2_N-BZM9** showed that this ligand binds two zones of the TvG6PD::6PGL enzyme, where the largest population of conformers was found, corresponding to the zone near the catalytic NADP^+^, with a binding energy of −7.56 kcal/mol, where (**R**)-**O_2_N-BZM9** formed an H-bond with the sulfoxide group and Arg239 (Figure 9E). Lastly, the other zone was the near the binding site of catalytic NADP^+^ with a binding energy of −7.81 kcal/mol, and the ligand formed one H-bond between benzimidazole ring and Asp357 and one H-bond with the nitro group and Asn221 (Figure 9G). Similar results were found with the (S) enantiomer of **O_2_N-BZM7** and **O_2_N-BZM9** compounds (Figure 9B,D,F,H). These results are related to the in vitro inhibition studies that were carried out with the recombinant enzyme of *T. vaginalis*, where **O_2_N-BZM7** revealed a *k_2_* value of 0.85 M^−1^·s^−1^, while **O_2_N-BZM9** had a *k_2_* value of 1.62 M^−1^·s^−1^. This indicates that the **O_2_N-BZM9** compound forms a TvG6PD::6PGL–inhibitor complex more quickly. Moreover, structural, and biochemical studies showed that **O_2_N-BZM9** affects the tertiary structure of the enzyme to a greater extent, decreasing 84% of intrinsic protein fluorescence with respect to native proteins, while **O_2_N-BZM7** led to a 30% reduction. 

### 2.5. In Vitro Assays over T. vaginalis Trophozoites

**Figure 10:**
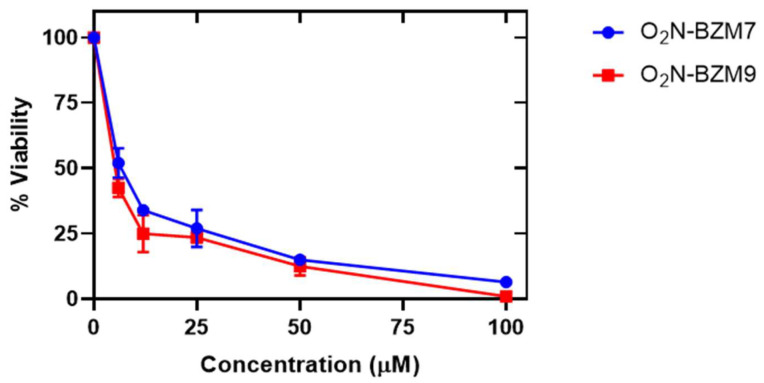
Antitrichomonal activity of the **O_2_N-BZM7** and **O_2_N-BZM9** compounds on *T. vaginalis* trophozoites’ viability. The trophozoites were incubated in the presence of compounds, by 24 h at 37 °C with increasing concentrations of each of the compounds, and the viability of the trophozoites was later determined. The values represent the mean ± standard deviation from three independent experiments; standard errors were lower than 5%.

The screening for the trichomonacidal activities of **O_2_N-BZM7** and **O_2_N-BZM9** using a wider range of concentrations showed the potential for compounds to reduce trophozoite viability by 100% at 100 μM after 24 h relative to the negative control. As expected, the 0.06% DMSO control did not affect trophozoite viability, motility, and morphology (trophozoites do not stain with trypan blue). The **O_2_N-BZM7** and **O_2_N-BZM9** compounds similarly affected trophozoite, reducing their viability by 52% at 6 µM, while 100% viability was lost at a concentration of 100 μM (Figure 10). Overall, the compounds decreased the number of trophozoites in strains of *T. vaginalis* in a dose-dependent manner. Thus, IC_50_ was determined as 6 μM and 4 μM for **O_2_N-BZM7** and **O_2_N-BZM9**, respectively. These values determined for *T. vaginalis* are lower than those determined for *G. lamblia* (Table 3), since, in a previous study, IC_50_ values of 14 μM and 17 μM were determined for **O_2_N-BZM7** and **O_2_N-BZM9**, respectively [11]. In addition, metronidazole, a drug currently used to treat giardiasis and trichomoniasis, was used as a positive control. As shown in Table 3, IC_50_ values of 12 μM and 4.8 μM were determined for *T. vaginalis* and *G. lamblia*, respectively. It is important to mention that **O_2_N-BZM7** and **O_2_N-BZM9** showed a lower IC_50_ value than metronidazole in *T. vaginalis*, while, in *G. lamblia*, metronidazole showed a lower IC_50_ value than the **O_2_N-BZM7** and **O_2_N-BZM9** compounds. Furthermore, the selectivity index (SI) demonstrates the differential activity of a pure compound: the greater the SI value, the more selective it is; thus, it is desirable to have a high SI, providing maximum antigiardial and trichomonicidal activities with minimal cell toxicity. On the basis of the SI, the data shown in Table 3 indicate that the compounds exhibit a high degree of selective toxicity in *G. lamblia* and *T. vaginalis* compared to metronidazole. We propose that **O_2_N-BZM7** and **O_2_N-BZM9** are considered for follow-up as antigiardial and trichomonicidal candidates with minimal cell toxicity.

*T. vaginalis* is the most common curable sexually transmitted disease worldwide, with a high incidence in women of reproductive age. However, persistent and recurrent infections by *T. vaginalis* are frequent, possibly due to the lack of timely diagnosis for this pathogen, as well as its resistance to prescription drugs [20]. Metronidazole and tinidazole are just two oral drugs that are used against trichomoniasis; hence, treatment options are lacking [21,22]. Several mechanisms may play a role in metronidazole resistance, including a decreased ability to reduce and activate 5-nitro-type prodrugs [23] with the detoxification of nitro radicals from the drug [24]. Therefore, the development of new agents suitable for the treatment of *T. vaginalis* is needed. There are several studies that support the efficacy of some compounds on *T. vaginalis*, such as ceragenins (cationic steroid antimicrobials compounds). However, with effective concentrations of 50 and 100 μM [25]. The other compounds are 2,8-bis(trifluoromethyl) quinoline analogs (QDA-1 and QDA-2) with an IC_50_ of 113.8 μM [26] and *N*-acylhydrazone derivatives [27]; two of them had IC_50_ values of 1.69 μM and 1.98 μM. These last compounds, as well as the **O_2_N-BZM7** and **O_2_N-BZM9** compounds proposed in this study, are promising antiparasitic compounds, since our results generally demonstrate the potential of **O_2_N-BZM7** and **O_2_N-BZM9** to affect both the enzymatic activity of TvG6PD::6PGL and the viability of *T. vaginalis* trophozoites, establishing a basis upon which to propose **O_2_N-BZM7** and **O_2_N-BZM9** as possible antitrichomonal and antigiardial drugs.

### 2.6. Pharmacokinetic Predictive Values

The physicochemical characteristics of drugs affect both the rate and the magnitude of absorption. Once the drug is dissolved in the fluids of the intestinal lumen, several physicochemical factors have a strong influence on its passive absorption, such as lipophilicity, solubility, pKa, and molecular size [28,29]. Therefore, a prediction of the absorption, distribution, metabolism, excretion, and toxicity (ADMET) parameters of compounds **O_2_N-BZM7** and **O_2_N-BZM9** was made using the platform ADMETLab 2.0 [30]. 

The in silico calculation, shown in Table 4, demonstrated that both compounds present high values of intestinal absorption, and only compound **O_2_N-BZM9** is able to cross the blood-brain barrier (BBB). The absorption parameters, using the MDCK (Madin–Darby canine kidney) and Caco-2 as predictive models of intestinal drug absorption, indicate that the compounds have a high passive permeability. The distribution parameters calculated were plasma protein binding and the volume of distribution. Both compounds present optimal values of distribution, the optimal plasma protein binding is less than 95%, the **O_2_N-BZM7** compound meets this parameter.

The oxidative metabolism by cytochrome P450 enzymes is the primary method used for the hepatic metabolism of drugs. The CYP2C19 and CYP3A4 isoforms participate in the metabolism of omeprazole [31]; therefore, the metabolic stability could be predicted, and compounds **O_2_N-BZM7** and **O_2_N-BZM9** are substrates of CYP2C19 and CYP3A4 isoforms. The excretion parameters were predicted, with the compounds showing satisfactory values of clearance and long half-lives (>3 h). Lastly, the toxicity parameters showed that the two main isoforms of CYP450 for **O_2_N-BZM7** and **O_2_N-BZM9** compounds showed low probabilities of being inhibited; therefore, the drug–drug interactions and undesirable adverse effects are likely to be low. Additionally, the compounds showed a very low prediction of hERG channel blockage and were considered non-cardiotoxic molecules (Table 4).

## 3. Materials and Methods

### 3.1. Purification of Fused GlG6PD:6PGLP and TvG6PD::6PGL Recombinant Proteins

The recombinant GlG6PD::6PGL and TvG6PD::6PGL proteins were expressed in the *E. coli* BL21(DE3)pLysS strain containing the plasmid pET3a-HisTEVP, which was cloned using the *g6pd*::*6pgl* gene from both *G. lamblia* and *T. vaginalis* [14,32]. The bacterial culture was grown in 2 L of Luria–Bertani (LB) medium supplemented with 100 µg/mL ampicillin (Sigma Aldrich, St. Louis, MO, USA) and kanamycin (50 µg/mL) for 12 h at 37 °C and 160 rpm. Thereafter, 0.3 mM of isopropyl-β-d-thiogalactoside (IPTG) was added to the culture at an optical density (OD 600 nm) of 0.8. The cells were pelleted by centrifugation, suspended in lysis buffer, and disrupted by sonication, as previously conducted by Morales-Luna et al. [14,30]. The crude extract was obtained by centrifugation at 10,000× *g* for 30 min at 4 °C, and the clear supernatants were incubated with a previously equilibrated Ni Sepharose high-performance column (equilibrium buffer: 50 mM KH_2_PO_4_, 150 mM NaCl, 5 µM NADP^+^, 2 mM DTT, and glycerol 10%; pH 7.35). The proteins were eluted with the same equilibrium buffer supplemented with 250 mM imidazole [14,32]. Then, the imidazole was removed from the proteins by consecutive dilutions using a microcon 10 kDa centrifugal filter unit (Millipore, Burlington, MA, USA). The purified proteins were digested with the Tobacco Etch Virus Protease (TEVP) to remove the (His)6-tag sequence located in the *N*-terminal region of both proteins. The purity of GlG6PD::6PGL and TvG6PD::6PGL proteins was analyzed by 12% SDS-PAGE gels and stained with colloidal Coomassie blue (R-250) (Sigma Aldrich, St. Louis, MO, USA). The purified proteins were used to perform both functional and structural assays in the presence and absence of selected hit compounds.

### 3.2. Activity Assay

The G6PD domain activity of the recombinant proteins was spectrophotometrically measured at 25 °C by monitoring the reduction in NADP^+^ at 340 nm [13]. The assay was performed with 1 mL of standard reaction mixture containing 0.1 M Tris-HCl, 0.01 M MgCl_2_, 1 mM NADP^+^, and 1 mM glucose-6-phosphate (G6P) at pH 8.3. The reaction was initiated with the addition of 1 µg/mL fused GlG6PD::6PGL or TvG6PD::6PGL enzymes.

#### 3.2.1. General Methodology for the Synthesis of Compounds

The five compounds were synthesized following a previously reported methodology, and the spectroscopic signals were accordingly with those previously published [11]. Briefly, the synthesis was carried out in two steps: first, the precursors were prepared with a mixture of 2-mercapto-5-benzimidazole and the substituted 2-chloromethyl pyridine in 1,2-dimethoxyethane (GLIMA) as the solvent and an alkaline medium (NaOH); the mixture was stirred for 8 h at 50 °C. The reaction was monitored by thin-layer chromatography (TLC) assay. The reaction mixture was extracted with CHCl_3_ (2 × 20 mL). The organic layer was dried over anhydrous Na_2_SO_4_ and concentrated under reduced pressure, and the residue was purified using column chromatography on silica gel, with CH_2_Cl_2_-hexane (3:1 *v*/*v*) as the eluent to give a pale-yellow solid. Second, the oxidation of the thioether group in precursors was carried out with 3-chloroperoxybenzoic acid and CHCl_3_ as a solvent; the peracid was added slowly, and the reaction was monitored every 5 min by TLC. After completion of the reaction, the mixture was treated with a saturated solution of sodium bicarbonate and extracted with CHCl_3_ (2 × 20 mL). The organic layer was dried over anhydrous Na_2_SO_4_, and the solvent was removed under reduced pressure. The residue was purified by means of column chromatography. 

#### 3.2.2. Determination of the Molar Extinction Coefficient of the Compounds

Each compound’s stock solutions were prepared to determine the compounds’ molar extinction coefficient (ε). Solutions for the spectroscopic measurements were prepared by immediately dissolving accurately weighed amounts of compounds in the appropriate solvent volume before running the spectra. The absorption spectra were recorded on a Cary 100 Bio UV/visible spectrophotometer (Walnut Creek, CA, USA) using a rectangular quartz cuvette with a path length of 1 cm, and an absorbance scan was performed in the wavelength range of 190 to 600 nm. To determine the ε of each compound, we worked with a group of solutions with concentrations of 5, 10, 20, 30, and 40 mg/mL, a range in which the changes in absorbance was linear (*r* = 0.9989). This allowed obtaining the absorbance results at a concentration of 1 mg/mL and obtaining the coefficients (Table 5).

### 3.3. In Vitro Screening of GlG6PD:6PGLP and TvG6PD::6PGL Inactivation with Compounds

Inactivation assays were performed with the compounds against GlG6PD::6PGL and TvG6PD::6PGL. Thus, all the compounds were activate immediately before the experiments as previously reported [3] and diluted using dimethyl sulfoxide (DMSO) as a solvent. For the assay, 0.2 mg/mL of the enzyme was incubated with the compounds at a concentration of 400 µM at 37 °C for 2 h. Following incubation, the activity was measured with a standard reaction mixture. Only the compounds that reduced the activity of the enzyme by more than 50% were used in the subsequent experiments.

The 50% inhibitory concentration (IC_50_) values of the **H-BZM2**, **O_2_N-BZM7**, and **O_2_N-BZM9** compounds against the fused enzymes from *G. lamblia* and *T. vaginalis* were established under the same conditions described above, with 0.2 mg/mL of the enzymes exposed for 2 h at compound concentrations ranging from 12 µM to 200 µM in TE buffer by 2 h at 25 °C. DMSO was maintained at 5% during incubation because this concentration did not affect the enzyme activity of both proteins. Then, aliquots from these incubations were withdrawn, and the residual activity was determined under standard conditions. The results are reported as the percentage of residual activity (using the activity of each enzyme incubated without any compound as 100% activity) versus the compound’s concentration.

### 3.4. Second-Order Rate Constant (k_2_) of Selected Hit Compounds 

The second-order rate constants of inactivation for each one of the compounds were calculated by obtaining the pseudo-first-order rate constant (*k_1_*) values. Pseudo-first-order inactivation rate constants (*k_1_*) were obtained at four or five fixed concentrations for each of the compounds. At the indicated times, aliquots were withdrawn to determine residual activity. Then, residual activity data were fitted using the monoexponential decay equation, as previously reported [11,13]. The previously obtained *k_1_* values were replotted against the concentrations of each compound, and the slope created by these plots corresponds to the second-order inactivation constant *k_2_* (M^−1^·s^−1^).

### 3.5. Spectroscopic and Chromatographic Characterization

#### 3.5.1. Circular Dichroism Experiments

To determine whether compounds caused changes in the secondary structure of the GlG6PD::6PGL and TvG6PD::6PGL proteins, we performed circular dichroism (CD) assays using a Jasco-810 spectropolarimeter (Jasco J-810^®^, Inc., Easton, MD, USA). Both proteins were adjusted at a protein concentration of 0.5 mg/mL with buffer P (50 mM phosphate, pH 7.35) and incubated for 2 h at 37 °C in the presence of the chemical compounds (IC_50_ of each compound). One control containing only enzyme was used in each assay. Then, the proteins were loaded in a quartz cell with a 0.1 cm path length, and spectral scans were recorded at 25 °C in ultraviolet circular dichroism (UV-CD) ranging from 200 to 260 nm. The spectra of the blanks (buffer P solution containing each of the compounds) were subtracted from all the obtained spectra that contained the protein. All the CD data were reported as molar ellipticity. The experiments were performed in triplicate.

#### 3.5.2. Intrinsic Fluorescence Assays

The effect of the compounds on the tertiary structure (3D) of the GlG6PD::6PGL and TvG6PD::6PGL proteins was evaluated by monitoring the changes in the intrinsic and extrinsic fluorescence using a Perkin-Elmer LS-55 spectrofluorometer (Perkin-Elmer, Wellesley, MA, USA). Both proteins were adjusted at 0.1 mg/mL in buffer P (50 mM phosphate, pH 7.35) and incubated with the IC_50_ concentration of the two synthesized benzimidazole compounds for 2 h at 37 °C. Following incubation, the proteins were excited at 280 nm, and we recorded the emission spectra from 310 to 500 nm with a scan speed of 150 nm/min using excitation and emission slits of 4 and 8 nm, respectively. The spectra of the blanks (buffer phosphate containing each of the compounds) were subtracted from all the obtained spectra that contained the protein, and the spectrum of the enzyme without the compound was used as control. The final spectrum for each compound was the average of five scans.

#### 3.5.3. Oligomeric Status of the Recombinant Proteins

To evaluate changes in the quaternary structure of the GlG6PD::6PGL and TvG6PD::6PGL proteins produced for the chemical compounds, a gel filtration column (GFC) analysis was performed. The proteins were adjusted at a final concentration of 0.5 mg/mL and incubated for 2 h at 37 °C with each one of the compounds. Then, the proteins were loaded on a Sephacryl TMS-200 HR HiPrepTM (16/60) gel filtration column (GE Healthcare, Chicago, IL, USA) previously equilibrated with phosphate buffer (50 mM pH 7.35) and coupled to the AKTA pure FPLC system (GE Healthcare). The same buffer was used as the mobile phase with a flow rate of 0.5 mL·min^−1^, while monitoring the absorbance at 280 nm (mUA). Moreover, the column was calibrated using a gel filtration standard kit (Bio-Rad, Tokyo, Japan). Both the proteins and the standard were performed in triplicate.

### 3.6. In Silico Docking Calculation 

The protein–ligand docking simulation was performed using the docking web service SwissDock [33], which is based on the protein-ligand docking program, EADock DSS. The models generated by Morales-Luna et al. 2018 [14] and Martinez-Rosas et al. 2022 [13] were used for the assay. The atomic coordinates of the models were submitted to the PDBsum server (PDBsum-EMBL-EBI) [34] to add the hydrogens to the structure. To identify all interactions on the proteins, blind docking was performed using the SwissDock Server (http://www.swissdock.ch/docking, accessed on 23 June 2022). The ligand structures of **O_2_N-BZM7** and **O_2_N-BZM9** (R) and (S) enantiomers were generated with Avogadro software 1.2.0 Qt version 4.8.6, and energy-minimized by UCSF Chimera software [35] and later docked on the models GlG6PD::6PGL and TvG6PD::6PGL. SwissDock generates all possible binding modes for each ligand; the most favorable binding modes for a given pocket are clustered. The predictions file provided the Cluster Rank/Element Full Fitness and estimated binding free energy ΔG. After molecular docking, we analyzed the best calculated binding poses, and the graphical representations were performed by PyMol Molecular Graphics System software (version 2.5.0, Schrödinger, LLC, New York, NY, USA).

### 3.7. In Vitro Assays over T. vaginalis Trophozoites

#### 3.7.1. *T. vaginalis* Culture 

The isolate *T. vaginalis*, Donne ATCC 30236 was axenically grown in trypticase–yeast extract–maltose (TYM) medium (pH 6.0), supplemented with 10% sterile horse serum (previously inactivated at 56 °C for 30 min), and incubated at 37 °C under microaerobic conditions [27,36]. The trophozoites were axenically maintained, and a trypan blue (0.4%) exclusion assay was performed to ensure that the minimum viability of 95% and logarithmic growth phase were achieved before proceeding to the antiparasitic assay.

#### 3.7.2. Anti-*T. vaginalis* Assay

Drug susceptibility assays were performed to analyze the trichomonacidal potential of **O_2_N-BZM7** and **O_2_N-BZM9** compounds against *T. vaginalis*, as previously described [27]. The compounds were diluted in dimethylsulfoxide (DMSO) as a vehicle for solubilization. The 50% inhibitory concentration (IC_50_) values against *T. vaginalis* were established with trophozoites exposed to benzimidazolic compounds for 24 h, with concentrations ranging from 6 μM to 100 μM. Trophozoites were adjusted to an initial density of 2.6 × 10^5^ trophozoites/mL of TYM medium. Then, 1.5 mL tubes were seeded with 150 mL of *T. vaginalis* trophozoites/tubes (2.6 × 10^5^ trophozoites/mL), **O_2_N-BZM7** and **O_2_N-BZM9** were added, and tubes were incubated at 37 °C with 5% CO_2_ for 24 h. After incubation, trophozoites were counted with trypan blue (0.4%) (1:1, *v*/*v*) on a Neubauer chamber to determine the trophozoites’ motility, morphology, and viability. Three controls were used in each assay: a negative control containing only trophozoites, a 0.06% DMSO control, and a positive control containing MTZ at 100 μM (Sigma-Aldrich, St. Louis, MO, USA). The IC_50_ was calculated using the GraphPad Prism 8.0 software Inc. version 8.0.2 (263) (San Diego, CA, USA).

## 4. Conclusions

The **O_2_N-BZM7** and **O_2_N-BZM9** compounds can inactivate the G6PD::6PGL enzyme from *G. lamblia* (GlG6PD::6PGL) and *T. vaginalis* (TvG6PD::6PGL), showing higher inactivation constants in both enzymes and an alteration of its tertiary structure. The main characteristic of these compounds is that they have a nitro group in position 5 of the benzimidazole ring, unlike to omeprazole compound. Furthermore, **O_2_N-BZM7** does not have substituents on the pyridine ring, while **O_2_N-BZM9** has a methyl and a trifluoroethoxy groups. According to the molecular docking findings, their nitro group was shown to be outstanding in the formation of hydrogen bonds with different amino acids of the G6PD domain. **O_2_N-BZM9** has a more favorable binding energy than **O_2_N-BZM7** due to its ability to form the enzyme–inhibitor complex more quickly than **O_2_N-BZM7**. As it does not bind to cysteines in the G6PD domain, the inactivation of the G6PD::6PGL fused enzymes is another important mechanism that is not found in the triosephosphate isomerase from *G. lamblia*. These findings reinforce the proposal of **O_2_N-BZM7** and **O_2_N-BZM9** as possible antigiardial and antitrichomonal drugs in clinical practice.

## Figures and Tables

**Table 1:** Percentage of enzyme activity of GlG6PD::6PGL and TvG6PD::6PGL after incubation at 37 °C for 2 h with benzimidazole derivative compounds of 400 μM.

Compounds	Inhibition (%) at 400 µM of GlG6PD::6PGL *	Inhibition (%) at 400 µM of TvG6PD::6PGL *
**H-BZM1**	62 ± 5	7 ± 5
**H-BZM2**	100 ± 5	47 ± 5
**H_2_N-BZM6**	100 ± 5	12 ± 5
**O_2_N-BZM7**	100 ± 5	95 ± 5
**O_2_N-BZM9**	100 ± 5	72 ± 5

* The results are relative to the control (enzyme without inhibitor).

**Table 2:** Summary of inhibition, IC_50_ values, and second-order rate constant values of inactivation (*k_2_*) of the compounds analyzed in this study.

Compounds	GlG6PD::6PGL			TvG6PD::6PGL		
	Inhibition (%)	IC_50_ (µM)	*k_2_* (M^−1^·s^−1^)	Inhibition (%)	IC_50_ (µM)	*k_2_* (M^−1^·s^−1^)
**H-BZM2**	100	24	3.3	47.5	-	-
**O_2_N-BZM7**	100	11	1.9	94.9	22	0.8
**O_2_N-BZM9**	100	15	6.1	71.9	240	1.6

**Table 3:** In vitro antigiardial and antitrichomonal activities of compounds, and the cytotoxic activity on Caco-2 cells.

Compound	O_2_N-BZM7 µM (SI)	O_2_N-BZM9 µM (SI)	Metronidazole µM (SI)
*T. vaginalis* IC_50_	6 (106)	4 (160)	12 (1.6)
*G. lamblia* IC_50_	14 (45)	17 (39)	4.8 (3.9)
Caco-2 (SI) CC_50_	640	663	19 [19]

**Table 4:** Pharmacokinetic predictive values calculated with ADMETLab 2.0 for compounds **O_2_N-BZM7** and **O_2_N-BZM9**.

		Compounds		
	Model	O_2_N−BZM7	O_2_N−BZM9	Comments
**A**	Human intestinal absorption	(+) High	(+) High	
	Caco-2 permeability	−4.509	−4.562	Optimal: higher than −5.15 log units
	MDCK permeability	0.00025	0.00018	High passive permeability: >2.0 × 10^−5^ cm/s
	Bioavailability (F)	>30%	>30%	
**D**	Volume distribution	0.338 L/kg	0.358 L/kg	Optimal: 0.04–20 L/kg
	BBB penetration	0.572	++	Probability of being BBB+
	Plasma protein binding	94	100	Optimal: <95%.
**M**	CYP2C19 substrate	(+) Yes	(+) Yes	
	CYP2C19 inhibitor	0.202	0.204	Probability of inhibition/blockage
	CYP3A4 substrate	(+) Yes	(+) Yes	
	CYP3A4 inhibitor	0.23	0.21	Probability of inhibition/blockage
**E**	Clearance (Cl)	2.908	4.026	mL/min/kg
	Half-life (T_1/2_)	>3 h	>3 h	
T	hERG blockers	0.024 inactive	0.018	Probability of inhibition
	Rat oral acute toxicity	−	−	

**Table 5:** UV molar extinction coefficients of compounds in aqueous phase. Percentage relative error < 5%.

Compound	Formula Weight (g/mol)	λ_max_ (nm)	ε (M^−1^·cm^−1^)
**H−BZM1**	257.31	299	15, 456
**H−BZM2**	315.39	299	17, 304
**H_2_N−BZM6**	384.37	306	13, 790
**O_2_N−BZM7**	302.30	345	17, 599
**O_2_N−BZM9**	414.35	345	14, 369

## Data Availability

Not applicable.

## Sample Availability

Samples of the compounds are available from the authors.
